# Different Repeat Annual Influenza Vaccinations Improve the Antibody Response to Drifted Influenza Strains

**DOI:** 10.1038/s41598-017-05579-4

**Published:** 2017-07-12

**Authors:** Ewan P. Plant, Lucy J. Fredell, Blake A. Hatcher, Xing Li, Meng-Jung Chiang, Martina Kosikova, Hang Xie, Olga Zoueva, Angelia A. Cost, Zhiping Ye, Michael J. Cooper

**Affiliations:** 1 Division of Viral Products, Center for Biologics Evaluation and Research, US Food and Drug Administration, Silver Spring, MD USA; 2 Sidwell Friends School, Washington, DC USA; 3 Chapelgate Christian Academy, Marriottsville, MD USA; 4 Armed Forces Health Surveillance Center, Silver Spring, MD USA

## Abstract

Seasonal influenza vaccine formulas change almost every year yet information about how this affects the antibody repertoire of vaccine recipients is inadequate. New vaccine virus strains are selected, replacing older strains to better match the currently circulating strains. But even while the vaccine is being manufactured the circulating strains can evolve. The ideal response to a seasonal vaccine would maintain antibodies toward existing strains that might continue to circulate, and to generate cross-reactive antibodies, particularly towards conserved influenza epitopes, potentially limiting infections caused by newly evolving strains. Here we use the hemagglutination inhibition assay to analyze the antibody repertoire in subjects vaccinated two years in a row with either identical vaccine virus strains or with differing vaccine virus strains. The data indicates that changing the vaccine formulation results in an antibody repertoire that is better able to react with strains emerging after the vaccine virus strains are selected. The effect is observed for both influenza A and B strains in groups of subjects vaccinated in three different seasons. Analyses include stratification by age and sex.

## Introduction

Influenza causes widespread seasonal disease and vaccination has been shown to reduce illness among the population^1–4^. Several studies have demonstrated that vaccination confers protection against virologically confirmed influenza^5^. However, understanding vaccination effectiveness is complicated because a large portion of the population is asymptomatically infected^6^. The circulating strains evolve from season to season leading to changes in vaccine formulation which further complicates comparisons between seasons.

Vaccine effectiveness can be measured by controlled clinical trials but comparisons between trials is complicated by factors such as the prevalence of the circulating virus and the closeness of virus match each season^7^. Observational studies, used in places where universal vaccination recommendations make it unethical to perform trials with placebos, are an increasingly common way of measuring vaccine effectiveness among the general population. Test-negative studies are a widely used method but the estimates for vaccine effectiveness vary widely depending on the study design and often have overlapping confidence intervals^8–10^. Correlates of protection, such as the hemagglutination inhibition (HI) titer, are widely used to assess vaccine efficacy. HI titers are associated with reduced risk of disease but there is significant variation in the protective titer depending on factors such as the age of the recipient and the severity of the influenza season^11–13^. It has also been reported that factors not detected in HI assays are associated with reduced risk of infection^14, 15^.

Changes in the annual vaccine formulation are compelled by the antigenic drift associated with seasonal influenza viruses. Despite the best efforts of a group of experts in selecting the candidate vaccine virus (CVV) strains to be included in the vaccine, there is often a mismatch between the CVV strains and circulating strains. The major decisions during the vaccine strain selection process are whether to retain the prior season’s CVVs or replace them with new CVVs better matched to the currently circulating strains. While most of the antibodies generated against the CVV are thought to target the head of the hemagglutinin (HA) there some antibodies that target the more conserved stem region^16, 17^. It is thought that sequential vaccinations with antigens that contain the same stem but different heads may boost the more broadly protective stem antibodies^18^. Indeed, repeated seasonal vaccination is associated with better health outcomes suggesting that vaccination, even with a mismatched vaccine, may boost cross-reactive antibodies^19, 20^ and interferon production^21^. But there are several reports that suggest regular annual vaccination results in lower vaccine effectiveness than just vaccination for the current season^22–25^. Here we used matched serum samples collected before and after sequential vaccinations with either the same vaccine or two vaccines with differing CVVs. We looked for differences in antibody titers toward the vaccine strains and strains that emerged post-vaccination using the hemagglutination inhibition (HI) assay. A trend for higher geometric mean titers (GMTs) is observed after sequential annual vaccination with different CVVs.

## Results

### Study Population

The serum samples used in this study come from a highly vaccinated group, consisting primarily of young adults in military service. Serum samples are collected upon entry to military service and at other times such as before and after deployment. The first sera sample from each individual was collected before a seasonal influenza vaccination and the matched sera sample was collected following a second seasonal influenza vaccination one year later. The average age of the subjects was 24.4 years (with a standard deviation of +/− 4.1). The study population was 24% female and 76% male. Three cohorts of subjects vaccinated during different influenza seasons were identified. The first cohort was subjects vaccinated around the time the 2009 pandemic H1N1 viruses emerged. The new pdmH1N1 viruses replaced the previously circulating seasonal H1N1 viruses and this change is referred to as antigenic shift. To reduce confounders none of the subjects received the monovalent vaccine that was available at that time. The second cohort was vaccinated during seasons where both of the H1N1 and H3N2 strains accumulated enough mutations to be become antigenically distinct from the earlier strains. This change is referred to as antigenic drift. The third cohort was vaccinated during seasons where the lineage of the influenza B strain included in the vaccine changed. Two groups of subjects were identified for each cohort; an ‘identical group’ vaccinated two years in a row with the same vaccine formulation, and a ‘differing group’ vaccinated once with the same formulation as the identical group and once with a different formulation. Each group contained matched pre- and post-vaccination samples from 80 subjects. The average age and ratio of males to females was similar in all the groups. The years each cohort was vaccinated, and the strains included in the vaccines, are presented in Table 1.

**Table 1:** Experimental Cohorts, Virus Vaccine Strains and Years Vaccinated.

Experimental Cohort	Vaccine Strains	Years Vaccinated
Drift Identical	A/Beijing/262/1995 (H1N1), A/Sydney/5/1997 (H3N2), B/Beijing/184/1993 (Yamagata)	1998 and 1999
Drift Differing	A/New Caledonia/20/1999 (H1N1), A/Moscow/10/1999 (H3N2), B/Beijing/184/1993 (Yamagata)	1999 and 2000
B Identical	A/New Caledonia/20/1999 (H1N1), A/Moscow/10/1999 (H3N2), B/Hong Kong/330/2001 (Victoria)	2002 and 2003
B Differing	A/New Caledonia/20/1999 (H1N1), A/Moscow/10/1999 (H3N2), B/Sichuan/379/1999 (Yamagata)	2001 and 2002
Shift Identical	A/California/7/2009 (pdmH1N1), A/Perth/16/2009 (H3N2), B/Brisbane/60/2008 (Victoria)	2010 and 2011
Shift Differing	A/Brisbane/59/2007 (H1N1), A/Brisbane/10/2007 (H3N2), B/Brisbane/60/2008 (Victoria)	2009 and 2010

The identical groups were vaccinated in both of the years indicated with the same vaccine formulation. The differing groups were vaccinated once with the same formulation as the accompanying Identical group and once with a different formulation. The differing vaccine strains and the year the different vaccine was administered are underlined.

### Using the emergence of the pandemic H1N1 virus to validate the approach

In this study we hypothesized that two different seasonal influenza vaccinations given one year apart would generate more cross-reactive antibodies than two identical vaccinations. Unlike many other influenza studies the timing of the sera sample collection was not linked to the timing of the vaccinations and the pre- and post-vaccination samples were collected more than one year apart. Because of these differences we chose a period of antigenic shift, where one circulating influenza strain is replaced by another antigenically distinct strain, to determine if differences in antibody levels could be distinguished using the hemagglutinin inhibition assay. The influenza B strains used in vaccinations during this time period did not change. The recipients in the differing group were vaccinated with the A/Brisbane/59/2007 (H1N1) antigen prior to the pandemic and with the A/California/07/2009 (pdmH1N1) antigen afterwards. Recipients in the identical group were vaccinated with the A/California/07/2009 (pdmH1N1) antigen two years in a row after the pandemic. Post-vaccination HI titers were measured for two H1N1 viruses and the B/Brisbane/60/2008 (Victoria) and A/Perth/16/2009 (H3N2) viruses which were also included in the vaccines after the pandemic (Table 2).

**Table 2:** HI Data for Pandemic Shift Cohort vaccinated in 2009–2011.

Antigen	V 1	V 2	Group	GMT (pre)	GMT (post)	Titer ≥ 40	Fold-change
A/Brisbane/59/2007 (H1N1)	−	−	Identical	45	86	57	1.9
	+	−	Differing	42	155	71	3.7
A/California/07/2009 (pdmH1N1)	+	+	Identical	187	326	78	1.7
	−	+	Differing	16	149	63	9.6
A/Perth/16/2009 (H3N2)	+	+	Identical	18	90	63	5.1
	−	+	Differing	16	51	48	3.1
B/Brisbane/60/2008 (Victoria)	+	+	Identical	63	304	79	4.8
	+	+	Differing	27	326	79	12.2

The names of the antigens used in the HI assay are listed in the first column. In the second and third columns + and − are used to indicate if the antigen was used in the first (V1) or second (V2) vaccination. The geometric mean titers prior to vaccination (pre) and after both vaccinations (post) are shown for each antigen for each group (Identical or Differing). The number of titers ≥40 is shown as a percentage. The fold-change is the average increase between the pre- and post-vaccination titers.

**Figure 1:**
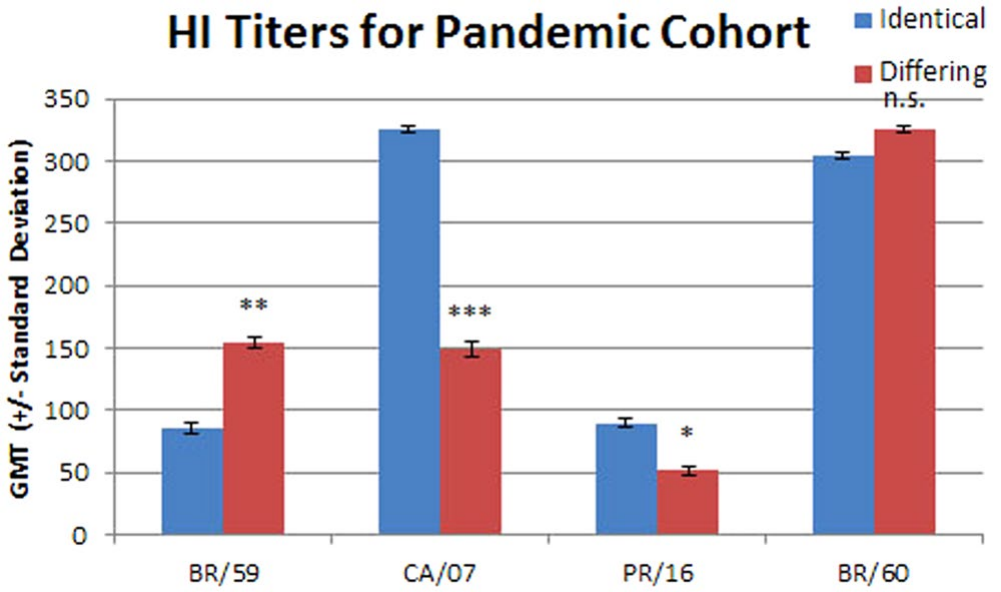
The geometric mean titers for sera from the identical and differing groups in the pandemic shift cohort are shown with standard deviations. The viruses used are listed on the x axis: BR/59, A/Brisbane/59/2007 (H1N1); CA/07, A/California/07/2009 (pdmH1N1); PR/16, A/Perth/16/2009 (H3N2); and BR/60, B/Brisbane/60/2008 (Victoria). The difference between the identical and differing groups was evaluated using the Mann Whitney rank test. n.s., not significant; *p ≤ 0.05; **p ≤ 0.01; and ***p ≤ 0.001.

Significant differences in HI titer between groups that received identical or differing vaccinations were observed when the antigen included in the vaccine changed. GMT titers toward the A/Brisbane/59/2007 (H1N1) virus were significantly higher in the differing group, which received one vaccination containing the A/Brisbane/59/2007 (H1N1) antigen, when compared to the identical group which did not receive an A/Brisbane/59/2007 (H1N1) vaccination (155 versus 86, P = 0.035) (Fig. 1). The GMT titers toward the A/California/07/2009 (pdmH1N1) virus were significantly higher in the identical group, which received two vaccinations with A/California/07/2009 (pdmH1N1), when compared to the differing group that only received one A/California/07/2009 (pdmH1N1) vaccination (326 versus 149, P = 0.0012). The identical group also received two A/Perth/16/2009 (H3N2) vaccinations compared to the one received by the differing group and the GMT was significantly higher in the identical group (90 versus 51, P = 0.012). There was no significant difference in the GMT titers toward the B/Brisbane/60/2008 (Victoria) virus which both groups were vaccinated against twice (304 versus 326 for the identical and differing groups respectively, P = 0.39).

Additional analyses on these data were performed to gauge the quality of the immune response. The percentage of samples with HI titers ≥ 40 was determined for each group (Table 2). 79% of both groups had HI titers ≥ 40 toward the B/Brisbane/60/2008 (Victoria) virus. The percentage was slightly lower for the A/California/07/2009 (pdmH1N1) and A/Brisbane/59/2007 (H1N1) viruses (78% and 57% respectively for the identical group and 63% and 71% respectively for the differing groups). 63% of the identical group and 48% of the differing group had HI titers ≥ 40 toward A/Perth/16/2009 (H3N2). The large proportion of the identical group with titers ≥ 40 toward A/Brisbane/59/2007 (H1N1) indicates that many in this group may have had prior exposure or vaccination with an A/Brisbane/59/2007 (H1N1)-like virus. Pre-existing antibodies specific to A/Brisbane/59/2007 (H1N1) should not be dramatically increased upon vaccination with the shifted A/California/07/2009 (pdmH1N1) antigen and the fold change in HI titer was used to assess this (Table 2). As expected, the average fold increase in A/Brisbane/59/2007 (H1N1) titers was less in the identical group, which received two sequential vaccinations with the A/California/07/2009 (pdmH1N1) antigen, than in the differing group that was vaccinated once with A/Brisbane/59/2007 (H1N1) (1.9 versus 3.7). Similarly the fold change for the A/Perth/16/2009 (H3N2) antigen was higher in the identical group, which was vaccinated twice with this antigen, than in the differing group, which was only vaccinated once (5.1 versus 3.1).

The pre-existing antibodies specific to A/California/07/2009 (pdmH1N1) would be expected to increase more in the identical group (receiving two vaccinations containing A/California/07/2009 (pdmH1N1)) than the differing group. However, the fold change toward the A/California/07/2009 (pdmH1N1) virus was much lower in the identical group than the differing group (1.7 versus 9.6) even though the GMTs were higher (Table 2). This result is not intuitive as revaccination is often associated with a boost in antibody levels. Pre-vaccination titers were compared to resolve this. The identical group had a higher pre-vaccination A/California/07/2009 (pdmH1N1) GMT than the differing group (187 versus 16 respectively, P < 0.0001). The pre-vaccination blood draws for the identical group occurred more than a year after the emergence of the pandemic H1N1 strain in early 2009. Thus, most of the subjects in the identical group were likely exposed to the pandemic strain prior to vaccination. Noone in this study received the monovalent pandemic vaccine that was available during this time. There was no difference in the pre-vaccination titers toward A/Brisbane/59/2007 (H1N1) for the identical and differing groups (45 versus 42 respectively, p = 0.85) or A/Perth/16/2009 (H3N2) (18 versus 16 respectively, p = 0.79).

It was also noted that although the GMTs and percentage of titers ≥ 40 toward the B/Brisbane/60/2008 (Victoria) are similar in both groups, there was a difference in fold change (12.2 and 4.8 for the identical and differing groups respectively). This too can be explained by the higher pre-vaccination titers of the identical group (63 versus 27, P < 0.0001) and suggests that those levels were boosted by an earlier vaccination or infection. As a visual aid to facilitate our interpretation of the HI data the HI titers from the pre- and post-vaccination sera were plotted (Supplementary Figure 1).

### Analyses for Influenza A virus strain changes

We next looked for seasons where antigenic drift of the influenza A viruses resulted in a change in the vaccine formulation. The northern hemisphere vaccine formulation remained the same for the 1998/99 and 1999/00 influenza seasons but both the H1N1 and H3N2 CVVs changed for the 2000/01 vaccine (Table 1). The identical group for the drift cohort received vaccines with the A/Beijing/262/1995 (H1N1) and A/Sydney/5/1997 (H3N2) antigens in the 1998/99 and 1999/00 seasons. The differing group was vaccinated with the same vaccine as the identical group for the 1999/00 season and then with a vaccine containing the A/New Caledonia/20/1999 (H1N1) and A/Moscow/10/1999 (H3N2) antigens for the 2000/01 season. The B/Beijing/184/1993 (Victoria) antigen was included in all the vaccines. HI titers toward the vaccine strains and strains that emerged later were determined (Table 3).

**Table 3:** HI Data for Drift Cohort vaccinated in 1998–2000.

Antigen	V 1	V 2	Group	GMT (pre)	GMT (post)	Titer ≥ 40	Fold-change
A/Beijing/262/1995 (H1N1)	+	+	Identical	26	228	71	8.0
	+	−	Differing	101	809	79	9.0
A/New Caledonia/20/1999 (H1N1)	−	−	Identical	14	58	49	4.3
	−	+	Differing	26	200	74	7.7
A/Brisbane/59/2007 (H1N1)	−	−	Identical	10	14	11	1.4
	−	−	Differing	152	63	55	4.1
A/California/07/2009 (pdmH1N1)	−	−	Identical	16	21	28	1.4
	−	−	Differing	16	45	50	2.9
A/Sydney/05/1997 (H3N2)	+	+	Identical	63	240	76	3.8
	+	−	Differing	73	331	79	4.5
A/Moscow/10/1999 (H3N2)	−	−	Identical	83	145	72	1.8
	−	+	Differing	65	219	78	3.4
A/Wyoming/03/2003 (H3N2)	−	−	Identical	14	45	49	3.3
	−	−	Differing	20	75	57	3.8
A/Perth/16/2009 (H3N2)	−	−	Identical	10	12	5	1.2
	−	−	Differing	10	12	3	1.2
B/Beijing/184/1993 (Victoria)	+	+	Identical	41	75	54	1.8
	+	+	Differing	22	81	56	3.7
B/Brisbane/60/2008 (Victoria)	−	−	Identical	23	57	51	2.5
	−	−	Differing	37	103	66	2.8

The names of the antigens used in the HI assay are listed in the first column. In the second and third columns + and − are used to indicate if the antigen was used in the first (V1) or second (V2) vaccination. The geometric mean titers prior to vaccination (pre) and after both vaccinations (post) are shown for each antigen for each group (Identical or Differing). The number of titers ≥40 is shown as a percentage. The fold-change is the average increase between the pre- and post-vaccination titers.

**Figure 2:**
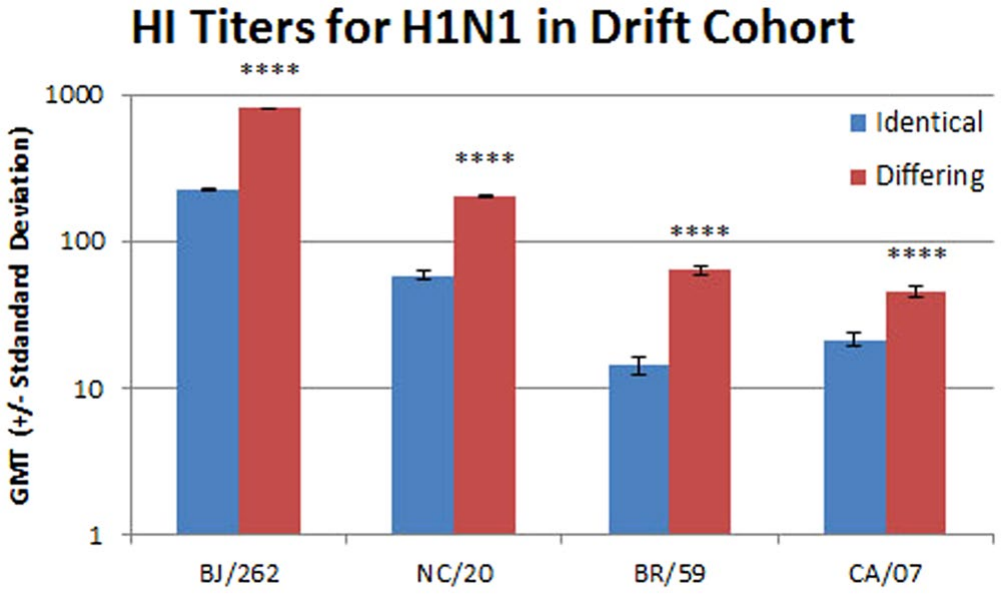
The geometric mean titers for sera from the identical and differing groups in the drift cohort for H1N1 viruses are shown with standard deviations. The viruses used are listed on the x axis: BJ/262, A/Beijing/262/1995 (H1N1); NC/20, A/New Caledonia/20/1999 (H1N1); BR/59, A/Brisbane/59/2007 (H1N1); and CA/07, A/California/07/2009 (pdmH1N1). The difference between the identical and differing groups was evaluated using the Mann Whitney rank test. ****p < 0.0001.

GMT titers toward the H1N1 viruses were significantly higher for the group vaccinated with two different formulations over two seasons (Fig. 2). Both the differing group and the identical group had robust GMT titers toward the A/Beijing/262/1995 (H1N1) strain (809 and 228 respectively, p < 0.0001). The GMT toward the A/New Caledonia/20/1999 (H1N1) strain, for which only the differing group was vaccinated with, was lower but still significantly different between the groups (200 and 58, p < 0.0001). The GMT titers for drifted and shifted H1N1 viruses, that emerged several years after the blood samples were drawn, were also significantly higher for the differing group (63 versus 14 for A/Brisbane/59/2007 (H1N1), p < 0.0001; and 45 versus 21 for A/California/07/2009 (pdmH1N1), p = 0.0001). The percentage of samples with titers ≥ 40 for was also higher in the differing group for all H1N1 viruses tested (Table 3).

The identical group received two vaccinations with A/Beijing/262/1995 (H1N1) antigen and the average fold change was slightly higher than the differing group which was only vaccinated once with the A/Beijing/262/1995 (H1N1) antigen (9-fold versus 8-fold, Table 3). A greater proportion of the identical group had lower pre-vaccination HI titers suggesting that in the intervening year, prior to the differing group pre-vaccination blood draw, many of the subjects received a vaccination containing, or were exposed to, a virus similar to A/Beijing/262/1995 (H1N1). Even though the identical group did not receive a vaccine containing the A/New Caledonia/20/1999 (H1N1) antigen, and a greater proportion had lower pre-vaccination titers, a 4.3 fold increase in titer was observed. This fits with the observation that repeated vaccination boosts titers to previously seen viruses^26^.

The increase in GMT, fold change and titers ≥ 40 toward the drifted and shifted viruses that emerged more than seven years after vaccination of these groups was analyzed. While only 2 subjects in the identical group had a pre-vaccination titer of ≥ 20 toward the shifted A/Brisbane/59/2007 (H1N1) strain, 15/80 subjects from the differing group had a titer of ≥ 20 (Supplementary Figure 2b) suggesting exposure to a similar antigen at that time. However, much of the increase in titer for the differing group was driven by the subjects that had pre-vaccination titers ≤ 10. The proportion of subjects with pre-vaccination HI titers of 20 toward the shifted A/California/07/2009 (pdmH1N1) strain was higher for both groups (Supplementary Figure 2b). However, fewer subjects in the identical group recorded an increase in post-vaccination titer and, like the data for the drifted A/Brisbane/59/2007 (H1N1) virus, most of the increase in titer was driven by subjects with pre-vaccination titers ≤ 10. This negative correlation between pre-vaccination titer and fold change was larger for the differing group for all H1N1 and H3N2 viruses tested.

**Figure 3:**
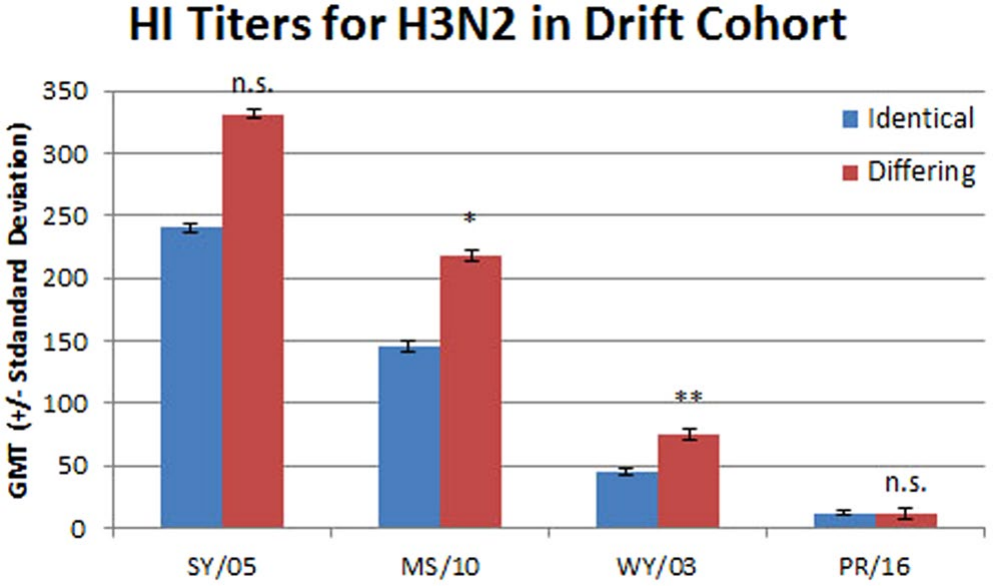
The geometric mean titers for sera from the identical and differing groups in the drift cohort for H3N2 viruses are shown with standard deviations. The viruses used are listed on the x axis: SY/05, A/Sydney/05/1997 (H3N2); MS/10, A/Moscow/10/1999 (H3N2); WY/03, A/Wyoming/03/2003 (H3N2); and PR/16, A/Perth/16/2009 (H3N2). The difference between the identical and differing groups was evaluated using the Mann Whitney rank test. n.s., not significant; *p ≤ 0.05; and **p ≤ 0.01.

Post-vaccination GMT titers toward the H3N2 vaccine strain viruses were higher in the differing group than in the identical group (Fig. 3). Only the differing group was vaccinated with the A/Moscow/10/1999 (H3N2) antigen and, as expected, the differing group GMT was higher than the identical group (219 versus 145, p = 0.059). Both groups were vaccinated with the A/Sydney/5/1997 (H3N2) antigen and, surprisingly, the GMT was higher in the group that received only one vaccination with that antigen (331 versus 240, p = 0.16). Again, this fits with the observation that vaccination boosts titers to previously seen viruses^26^. The differing group also had a greater percentage of subjects with titers ≥ 40 and a larger fold change toward each H3N2 antigen used in the vaccines (Table 3). When the A/Perth/16/2009 (H3N2) virus, a virus that emerged a decade after the vaccinations, was tested most post-vaccination HI titers were ≤ 10 for both groups. This indicates that no cross-reactive H3N2 antibodies were present in either group.

The difference in response toward drifted H1N1 viruses and drifted H3N2 viruses caught our attention. Possible reasons for this include differences in antigenic distance. In the years between the inclusion of the A/New Caledonia/20/1999 (H1N1) and A/Brisbane/59/2007 (H1N1) strains in the vaccine there was only one other H1N1 strain recommended by the WHO. In contrast, between A/Moscow/10/1999 (H3N2) strain and A/Perth/16/2009 (H3N2) there were four different H3N2 strains recommended for inclusion in the vaccine. It has also been noted that H3N2 viruses evolve more rapidly than the other lineages^27^. To address this, the serum samples were tested with the A/Wyoming/03/2003 (H3N2) virus (Fig. 3 and Table 3). The GMT for the differing group was significantly higher than that of the identical group (75 versus 45, p = 0.022). The differing group also had a greater percentage of subjects with titers ≥ 40 compared to the identical group (57% versus 49%). Both groups had fold change values toward the A/Wyoming/03/2003 (H3N2) antigen below 4 (3.8 and 3.3).

Both the differing and identical groups were vaccinated two years in a row with formulations containing B/Beijing/184/1993 (Victoria). The sera were tested for HI titers toward the B/Beijing/184/1993 (Victoria) antigen (Table 3 and Supplementary Figure 2a). There was no significant difference in GMT values between the groups (75 versus 81, p = 0.78). The two groups had a similar percentage of subjects with titers ≥ 40 (54% and 56%) but the differing group had a larger fold change toward the B/Beijing/184/1993 (Victoria) antigen (3.7 versus 1.8).

### Analyses for Influenza B virus strain changes

**Figure 4:**
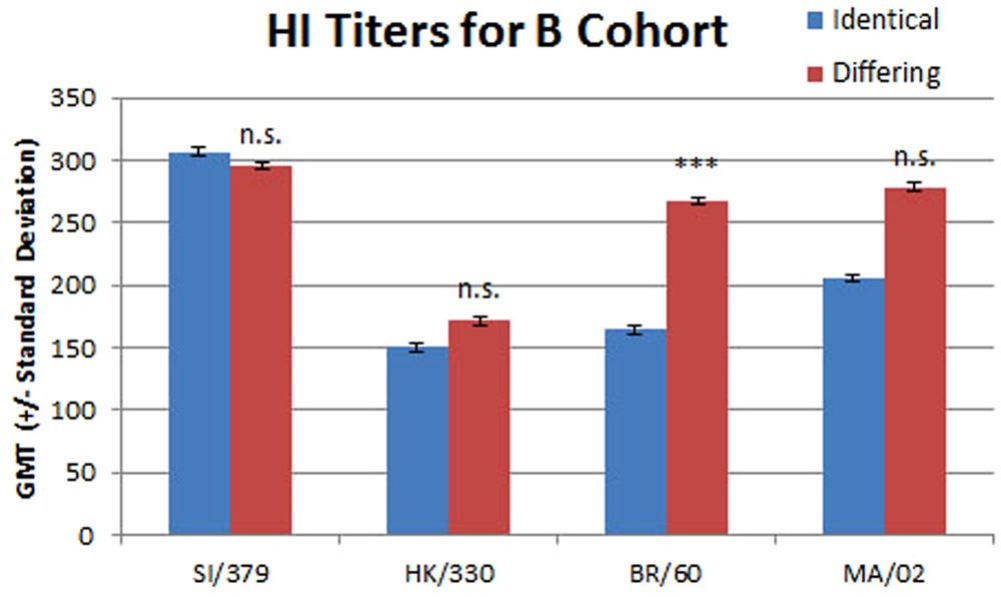
The geometric mean titers for sera from the identical and differing groups in the B virus cohort are shown with standard deviations. The viruses used are listed on the x axis: SI/379, B/Sichuan/379/1999 (Yamagata); HK/330, B/Hong Kong/330/2001 (Victoria); BR/60, B/Brisbane/60/2008 (Victoria); and MA/02, B/Massachusetts/02/2012 (Yamagata). The difference between the identical and differing groups was evaluated using the Mann Whitney rank test. n.s., not significant; and ***p ≤ 0.001.

Sera from different seasons were selected for the analysis of HI titers toward influenza B viruses. The northern hemisphere vaccine formulation remained the same for the 2002/03 and 2003/04 influenza seasons but the B strain differed in the earlier 2001/02 vaccine (Table 1). The identical group for this experiment was vaccinated for the 2002/03 and 2003/04 influenza seasons with a vaccine containing the Victoria lineage B/Hong Kong/330/2001 antigen. The differing group was vaccinated with a vaccine containing the Yamagata lineage B/Sichuan/379/1999 (Yamagata) antigen in the 2001/02 season followed by the same vaccine as the identical group for the 2002/03 season. The A/New Caledonia/20/1999 (H1N1) and A/Moscow/10/1999 (H3N2) antigens were included in each of the three seasons. HI titers toward the two influenza B vaccine strains and one strain each from the Victoria and Yamagata lineages that emerged later were determined (Table 4 and Fig. 4).

**Table 4:** HI Data for B Experimental Cohort vaccinated in 2001–2003.

Antigen	V 1	V 2	Group	GMT (pre)	GMT (post)	Titer ≥ 40	Fold-change
B/Sichuan/379/1999 (Yamagata)	−	−	Identical	118	306	76	2.6
	+	−	Differing	90	296	80	3.3
B/Hong Kong/330/2001 (Victoria)	+	+	Identical	20	151	75	7.7
	−	+	Differing	18	171	74	9.7
B/Massachusetts/02/2012 (Yamagata)	−	−	Identical	101	206	76	2.0
	−	−	Differing	72	279	79	3.9
B/Brisbane/60/2008 (Victoria)	−	−	Identical	19	164	75	8.7
	−	−	Differing	29	267	78	9.4
A/New Caledonia/20/1999 (H1N1)	+	+	Identical	70	182	75	2.6
	+	+	Differing	53	127	71	2.4
A/Moscow/10/1999 (H3N2)	+	+	Identical	63	207	74	3.3
	+	+	Differing	71	190	71	2.7

The names of the antigens used in the HI assay are listed in the first column. In the second and third columns + and − are used to indicate if the antigen was used in the first (V1) or second (V2) vaccination. The geometric mean titers prior to vaccination (pre) and after both vaccinations (post) are shown for each antigen for each group (Identical or Differing). The number of titers ≥ 40 is shown as a percentage. The fold-change is the average increase between the pre- and post-vaccination titers.

There was no significant difference in the GMT for the group receiving two vaccinations and the group receiving one vaccination with the B/Hong Kong/330/2001 (Victoria) antigen (306 versus 292, p = 0.25; Fig. 4). There was no significant difference in GMT for the B/Sichuan/379/1999 (Yamagata) virus between the group vaccinated once with that antigen and the group that did not receive that antigen (171 versus 151, p = 0.34). In line with the observation that HI titers to previously circulating viruses increase with vaccination the group not vaccinated with the B/Sichuan/379/1999 (Yamagata) antigen had robust post-vaccination titers after vaccination with the B/Hong Kong/330/2001 (Victoria) antigen^26^. The GMT toward strains that were not circulating at the time of vaccination is higher in the differing group for both the Yamagata lineage strain B/Massachusetts/02/2012 (Yamagata) (279 versus 206, p = 0.11) and the Victoria lineage strain B/Brisbane/60/2008 (Victoria) (267 versus 164, p < 0.0001). The titers toward the Yamagata lineage strain were higher than those toward the Victoria lineage strain. The proportion of subjects with HI titers ≥ 40 was higher than 70% across all groups for all four viruses (Table 4). However, the fold change was higher for the differing group for all four viruses tested.

Both the differing and identical groups were vaccinated two years in a row with formulations containing A/New Caledonia/20/1999 (H1N1) and A/Moscow/10/1999 (H3N2). The sera were tested for HI titers toward these antigens. There was no significant difference in GMT values between the groups for the A/Moscow/10/1999 (H3N2) (207 versus 190, p = 0.77) virus but a lower GMT was observed for the differing group for the A/New Caledonia/20/1999 (H1N1) virus (182 versus 127, p = 0.0496) (Supplementary Figure 3a). The two groups had more than 70% of subjects with titers ≥ 40 (Table 4).

### Comparison of Sera from Different Years

Subjects from the B virus and drift cohorts were all vaccinated with the A/New Caledonia/200/1999 (H1N1) and A/Moscow/10/1999 (H2N3) antigens. Post-vaccination GMT titers were higher in the more recent years for identical groups for both A/New Caledonia/20/1999 (H1N1) (58, 1999; 182, 2003) and A/Moscow/10/1999 (H3N2) (146, 1999; 207, 2003) (Tables 3 and 4). This increase by year was not observed for the differing groups for either the H1N1 (200, 2000; 127, 2002) or H3N2 (219, 2000; 190, 2002) virus. However the differing group GMT values were higher than the identical group values in each experiment and the difference was significant for both antigens in the drift cohort.

We assessed the HI titer for all serum samples used in all cohorts toward the B/Brisbane/60/2008 (Victoria) virus. Only the subjects in the pandemic shift cohort were vaccinated with this antigen as it emerged several years after the sera were collected for the B virus and drift cohorts (Table 1). There was some reactivity toward B/Brisbane/60/2008 (Victoria) in all groups that were boosted upon vaccination (Tables 2, 3 and 4) suggesting exposure to a similar antigen at the earlier time points. The HI titers from post-vaccination sera were higher in the more recent years for both the identical groups (57, 1999; 164, 2003; 304, 2011) and differing groups (103, 2000; 267, 2002; 326, 2010). The differing group values were slightly higher whether the sera were collected in the year prior to or the year after the identical group sera was collected.

### Correlations with Participant Characteristics (medical encounters, age, and sex)

Antibody levels are known to be increased by both vaccination and infection and many infections result in medically attended respiratory illness^28, 29^. To ensure that the effects on HI titer observed in this study were not unduly affected by infection, subjects who had a medical encounter designated as ILI or PI were identified. 131 of the 480 subjects had a medical encounter between blood draws (27%). A larger number were in the differing groups (n = 75, 31%) than the identical groups (n = 56, 23%). The differences in the GMT between the differing and identical groups remained significant for all of the antigens tested in the pandemic shift cohort except for the A/Perth/16/2009 (H3N2) antigen (Supplementary Figure 4). The GMT was higher for the identical group when all subjects were considered (90 versus 51, p = 0.012) but lower when the medically attended subjects were removed (10 versus 80, p = 0.16). The differences between the identical and differing groups remained significant in the drift cohort for all antigens except one. Although the GMT remained higher for the differing group with the A/Wyoming/03/03 (H3N2) antigen the difference no longer reached significance (45 versus 75, p = 0.022 for all; 47 versus 72, p = 0.13 for not medically attended). In the B cohort the GMT value for the A/New Caledonia/20/1999 (H1N1) antigen was significant for the entire population (182 versus 127, p = 0.0496) but lost significance (202 versus 131, p = 0.0501) when medically attended encounters were excluded. The non-significant post-vaccination differences for other antigens in the B cohort remained non-significant but for the B/Hong Kong/330/2001 (Victoria) and A/Moscow/10/1999 (H3N2) antigens the group with the higher titer was reversed.

Antibody levels are known to wane over time. The experimental setup for this project used a broad time range for the post-vaccination serum collection; serum samples were collected between 21 days and six months after the second vaccination. An analysis was performed to determine if there was a correlation between post-vaccination HI titers and the length time after vaccination that the serum was collected.

Most of the correlations between post-vaccination titer and sera collection date were negative (a lower HI titer the later the sera collection date) (Supplementary Table 1). There was one significant negative correlation for one antigen in the pandemic shift cohort in one group (B/Brisbane/60/2008 (Victoria) antigen (−0.28, p ≤ 0.01) for the identical group). Five of the seven remaining correlations for the pandemic shift cohort were also negative but did not reach significance. In the drift cohort there was a negative, but non-significant, correlation for the influenza vaccine strains A/Beijing/262/1995 (H1N1), A/New Caledonia/20/1999 (H1N1), A/Sydney/5/1997 (H3N2), A/Moscow/10/1999 (H3N2) and B/Beijing/184/1993 (Victoria). There was a negative, but non-significant, correlation for the influenza B vaccine strains in the B virus cohort with the exception of B/Sichuan/379/1999 (Yamagata) in the differing group (0.228, p ≤ 0.05). There was a negative, but non-significant, correlation for the influenza A vaccine strains (A/New Caledonia/20/1999 (H1N1) and A/Moscow/10/1999 (H3N2)) in the identical group but not for the differing group in the B virus cohort. In total, 30/40 correlations were negative (Supplementary Table 1).

The immune response of pediatric, adult and elderly populations differs. The age range of study subjects in this study is relatively small with 443/480 (92%) aged between the 19 and 30 at the time of the first vaccination. The data was analyzed for a correlation between age and HI titer (Supplementary Table 2). There was a negative correlation (a lower HI titer the older the subject) for the influenza A strains in the pandemic shift cohort. The correlation was significant for the A/California/07/2009 (pdmH1N1) strain for both groups (−0.249, p ≤ 0.01 for the identical group and −0.282, p ≤ 0.01 for the differing group).

The correlation between age and HI titer was negative for the H1N1 strains used in the drift cohort but only reached significance for the A/New Caledonia/20/1999 (H1N1) strain (−0.241, p ≤ 0.05 for the identical group and −0.227, p ≤ 0.05 for the differing group). The negative correlation reached significance for three of the H3N2 strains in the identical group (−0.380, p ≤ 0.001 for A/Sydney/5/1997 (H3N2); −0.339, p ≤ 0.001 for A/Moscow/10/1999 (H3N2); and −0.306, p ≤ 0.01 for A/Wyoming/03/2003 (H3N2)) but not in the differing group (Supplementary Table 2).

In the B virus cohort there were negative correlations between age and HI titer for the A/New Caledonia/20/1999 (H1N1) and A/Moscow/10/1999 (H3N2) antigens which were included in both vaccinations for all subjects. This reached significance for the H1N1 virus in both groups (−0.317, p ≤ 0.05 for the identical group and −0.519, p ≤ 0.001 for the differing group) and for the H3N2 virus in the differing group (−0.310, p ≤ 0.01). There was also a negative correlation for the B/Sichuan/379/1999 (Yamagata) which was included in the first vaccination for the differing group (−0.239, p ≤ 0.01). The correlation did not reach significance for the B/Hong Kong/330/2001 (Victoria) antigen in either the identical or differing groups. There were negative correlations between age and post-vaccination titer for 33/40 comparisons with 13 of these reaching significance (Supplementary Table 2).

Higher post-vaccination antibody levels were unexpectantly correlated with older subjects for some antigens. For example, a positive correlation between age and HI titer for B/Brisbane/60/2008 (Victoria) reached significance for identical group in the pandemic experimental group (0.335, p ≤ 0.01). However, there was also a significant correlation between age and pre-vaccination titers (0.259, p ≤ 0.05). The difference between the pre- and post-vaccination HI titers (fold change) was used to further analyze this. The correlation between age and fold change for B/Brisbane/60/2008 (Victoria) was negative, but not significant (−0.032, p = 0.8). An analysis of the correlation between HI titer and fold change was performed for the other antigens (Supplementary Table 2). A significant negative correlation for the A/Brisbane/59/2007 (H1N1) antigen was observed for the differing group (−0.290, p ≤ 0.01) in the pandemic shift cohort. A significant negative correlation between age and fold change for the H1N1 vaccine antigens was also observed in the drift cohort for differing group (−0.238, p ≤ 0.05 for A/Beijing/262/1995 (H1N1) and −0.222, p ≤ 0.05 for A/New Caledonia/20/1999 (H1N1)) but not the identical group. The correlations for the H3N2 antigens did not reach significance. There were negative correlations between age and fold change for the differing group in the B virus cohort but the correlation only reached significance for the A/New Caledonia/20/1999 (H1N1) virus. In the B virus cohort there were non-significant positive correlations for the B viruses in the identical group and non-significant negative correlations for the A viruses. There were negative correlations between age and fold change for 31/40 comparisons with 5 of these reaching significance (Supplementary Table 2). This is in line with earlier work demonstrating that higher pre-vaccination titers are associated with lower fold change as age increases^30^.

The immune response of male subjects differs from females. 113 of the 480 subjects were female (24%). There were more female subjects in the differing groups (n = 63, 26%) than the identical groups (n = 50, 21%). There was no significant difference in the average age or timing of the sera sampling relative to the vaccination between the male and female subjects.

The GMT values between men and women were compared. For most antigens the trend for a higher GMT in either the identical or differing group remained the same for the subpopulations considered but in some instances the significance differed (Supplementary Table 3). For example, the GMT values for the A/California/07/2009 (pdmH1N1) antigen in the pandemic shift cohort were significantly different for women (338 for identical and 87 for differing, p = 0.019) but not for men (323 for identical and 191 for differing, p = 0.058). The only antigen for which the GMT was higher in the opposite groups for women and men was the B/Beijing/184/1993 (Victoria) antigen in the drift cohort. Both the identical and differing groups were vaccinated twice with this antigen and there was no significant difference between the post-vaccination GMTs of the identical and differing groups for the whole population or the two subpopulations considered separately. The GMT was lower in the differing group for women (59 versus 39, p = 0.37) and higher in the differing group for men (81 versus 102, p = 0.36). The GMT for the B/Beijing/184/1993 (Victoria) antigen was significantly higher for men when compared to women in the differing groups (102 versus 39 respectively, p = 0.014) but not the identical groups (81 versus 89 respectively, p = 0.56).

One significant difference in the post-vaccination GMT between men and women was observed in the B experimental cohort. The A/New Caledonia/20/1999 (H1N1) antigen for the differing group was higher for men (150 versus 71, p = 0.032). The trend between identical and differing groups remained the same but the difference was significant for the female subjects (199 versus 71, p < 0.004) or all subjects (182 versus 127, p = 0.041) but it was not significant for men alone (177 versus 150, p = 0.38). The post-vaccination values were lower for women in 23/40 comparisons (Supplementary Table 3).

The trend for lower titers for women was also observed in the pre-vaccination GMT values. The pre-vaccination GMT values were lower for women in 15/20 identical groups and 14/20 differing groups (data not shown). The GMT values of the pre-vaccination titers for four antigens were significantly lower for the identical groups in two cohorts. Those were the A/Beijing/262/1995 (H1N1) and A/New Caledonia/20/1999 (H1N1) antigens in the drift cohort and the B/Sichuan/379/1999 (Yamagata) and B/Hong Kong/330/2001 (Victoria) antigens in the B cohort. The GMT pre-vaccination titer for the differing group was significantly lower for the B/Beijing/184/1993 (Victoria) antigen in the drift cohort.

## Discussion

There are conflicting reports about the effect of repeated annual vaccination on the immune response. Some studies have indicated that receipt of the prior season vaccine dampers the response to the current season vaccine^31–33^. This has been suggested as a reason for lower vaccine effectiveness in some studies^22–25^. Conversely it has been reported that infection or vaccination increases the pre-existing antibody levels^26, 34^.

Different hypotheses have been proposed to account for the difference in repeated annual influenza vaccination response. Smith *et al*., proposed that pre-existing cross-reactive antibodies generated in response to prior vaccinations might mute the generation of antibodies to the new vaccine if the antigens were similar enough^35^. A recent study by Ellebedy *et al*., reported that vaccination might not increase the somatic mutation frequency in B-cells as induced B-cells originated from pre-existing memory B cells which already had receptors of high affinity^36^. He *et al*., observed that repeated immunization with inactivated A(H1N1)pdm09 vaccine resulted in reduced vaccine-specific and cross-reactive plasma-blast derived antibody responses^37^. As a corollary to these studies cross reactive H3N2 antibodies have been described in sera collected just after periods of marked antigenic drift^38^.

A person’s history of exposures and vaccination affects the antibody landscape. Broadly neutralizing antibodies increase with age^39^ and vaccination plays a role in this. For example, antibodies that neutralize influenza viruses of animal origin are present in higher titers in subjects that have received multiple vaccinations^40, 41^. The serum samples analyzed in this work are from a highly vaccinated population. It has been reported elsewhere that vaccination with strains that are not well matched can still result in good immune responses. For example, vaccination with the seasonal TIV has previously been shown to be associated with increased A(H1N1)pdm09 antibody responses^42–44^. The number of laboratory confirmed A(H1N1)pdm09 influenza infections was also lower in recipients of a seasonal TIV in a placebo controlled trial^7^. Fonville *et al*., noted that vaccination with an H3N2 strain for which the population had less immunity resulted in a significantly greater responses toward later antigenic clusters than vaccination with a strain for which there had been some prior exposure. In addition, the back-boost provided by the more distant vaccine strain was similar to that provided by a strain for which pre-existing immunity was present^26^.

Here we show that vaccine formulation plays a significant role in generating antibodies toward drifted viruses. Our data demonstrates that sequential seasonal vaccinations with different formulations results in higher HI titers toward drifted strains than two vaccinations with the same formulation. This effect was observed for H1N1, H3N2 and B virus strains during different influenza seasons suggesting that this result is not specific to one antigen or season. One caveat of this study is that only subjects who received inactivated trivalent vaccines were considered. The study did not include live attenuated vaccines or vaccines that contain both Yamagata- and Victoria-lineage B virus strains.

The rate and frequency of antigenic change varies between virus subtypes and this is reflected by differences in antigenicity^27, 45, 46^. The HI data presented here indicates that the magnitude of cross-reactive antibodies generated by differing sequential vaccinations might depend on antigenic distance between the strains included in the sequential vaccines. The drift experimental cohort was vaccinated with H1N1 and H3N2 viruses isolated in the late 1990’s. The H1N1 viruses (A/Beijing/262/1995 and A/New Caledonia/20/1999) are from different antigenic clusters whereas the H3N2 viruses (A/Sydney/05/1997 and A/Moscow/10/1999) are part of the same cluster^27^. Highly significant differences in the response toward the H1N1 viruses were observed between the identical and differing groups but the difference was less significant or not significant for the H3N2 viruses tested. This fits with the antigenic distance model proposed by Smith *et al*.^35^, but it remains unclear the extent of antigenic distance required for the different virus types.

We considered the role infection may have in our analysis. Natural infection in children results in significantly higher GMT, seroconversion and seroprotection rates after subsequent vaccination compared to non-infected vaccinees^47^. Natural infection leads to in greater B-cell responses to NP and heterosubtypic HAs^37^. Members of the military are required to see a health care provider in order to report in sick. Removing data from subjects who presented with ILI or PI did not affect the outcome of this study. However, in our proof of concept experiment we noted that the pre-titers of the differing group toward the A(H1N1)pdm09 virus were significantly higher than the pre-titers for the identical group. For one influenza vaccine trial it has been reported that placebo subjects had high antibody titers toward the A(H1N1)pdm09 virus even though they did not receive a vaccination or present with influenza-like illness^48^. We noted the increase in HI titers toward the B/Brisbane/60/2008 (Victoria) antigen in the years leading up to its isolation suggesting similar viruses may have circulated during this period. These data suggest that subclinical infections could be a major source of influenza antibodies.

The study design does not account for antibodies due to subclinical infections and this could have hampered comparisons between the identical and differing groups. The group with the later collection dates may have higher pre-vaccination titers due to exposure to viruses that circulated during the intervening period. This was observed for the A/California/07/2009 (pdmH1N1) antigen in the antigenic shift cohort. However, limiting analyses to samples with low pre-vaccinations titers (≤20) did not alter the trends observed for the shift cohort (data not shown). Additionally, as shown for the drift cohort where a larger number of antigens were tested, the post-titer GMTs were consistently higher for more subjects in the differing group no matter which group had the more subjects with higher pre-GMT titers (Supplementary Figure 2a,b and c). This trend held true for virus strains used in the vaccine and viruses that emerged several years later.

Sex-based differences in immune responses toward influenza vaccinations are known. Evidence of a more robust immune response in women three weeks post-vaccination has been demonstrated^49, 50^ and it has been reported that men are more affected by seasonal influenza^51^. In contrast, weaker antibody avidity has been reported for women younger than 65 years compared to men^52^. The health status and virus strain also affects antibody titers. Pregnant women younger than 25 years were more likely to maintain protective titers toward influenza A viruses, but not influenza B virus, than women ≥25 years old^53^. Here we report lower GMT values in women both before and after vaccination for several antigens. Interestingly, most of these observations were with pre-vaccination titers suggesting a sex difference in antibody waning.

Protective antibodies have been shown to persist for more than a year in different populations^48, 53–56^. But vaccine effectiveness may be lost during the latter six months of the season^57^. The data reported here demonstrates that annual influenza vaccination boosts HI titers toward strains represented in the vaccine formulation. For all groups and antigens analyzed the average fold change in HI titer was greater than 1. However the experimental design only included one serum sample before the first vaccination and one serum sample after the second vaccination so we could not analyze the impact of each vaccination individually by HI assay. The lack of different time points also precluded a thorough analysis of antibody waning.

In conclusion, we demonstrate that repeated annual influenza vaccination increases HI titers in a highly vaccinated population. Greater increases in titer were observed when the strains in the vaccine formulation had changed and this also resulted in more broadly reactive antibodies. Medically attended influenza infections or ILI did not influence this result but subclinical infections likely contribute significantly toward the antibody landscape.

## Methods

### Serum Sample Criteria

Serum samples were obtained from the Department of Defense Serum Repository in accordance with the relevant guidelines and regulations. The following were the requirements for the inclusion of serum samples. The vaccinations occurred between September 1st and December 15th and the individual received only one influenza vaccination per year. Individuals did not receive the monovalent 2009 pandemic vaccine. The vaccine used was an inactivated influenza vaccine not a live attenuated vaccine. The individual was an active component service member in the service for the entire time period of the group vaccination window they were selected for. To reduce potential confounders the length of time in the military was restricted to less than 10 years before the post-vaccination sample, and medical encounter data for influenza-like illness (ILI) and pneumonia and influenza (PI) for the time period of the study was included.

The pre-vaccination serum collection window is within the six month period prior to vaccination. Antibody levels peak 2-3 weeks post-vaccination and wane over time. The post-vaccination collection window is greater than 21 days post-vaccination and less than 6 months after vaccination. The identical and differing groups were matched, where possible, with regard to the timing of the blood draws.

### Hemagglutination Inhibition Assays

HI assays were performed using standard methods. Each volume of serum was treated with 3 volumes of receptor destroying enzyme (Accurate Chemical, NJ) at 37 °C overnight, which was then inactivated by heating in a 56 °C water bath for 30 minutes. Six volumes of PBS were added to make the final sera concentration of 1:10. After RDE treatment 50 microliters of each diluted serum was added to the first well of a 96-well plate. 25 microliters of PBS was added to the next 11 wells and serial 1:2 dilutions of the sera were made using robotic diluters. 25 microliters of virus titrated to a final HA titer of 8 per was added to the first 11 wells and the plates incubated at room temperature for 30 minutes. The last well was left as a no-sera control. Freshly diluted turkey red blood cells (0.5%) for H1N1 and B antigens, or guinea pig red blood cells (1.0%) for H3N2 antigens, were added to all wells and the plates incubated for an additional hour. After incubation the last dilution of sera that completely inhibited agglutination was recorded.

### Data Analysis

All data were included in the analyses. Geometric mean titers (GMT) and fold change were calculated from the log values of the HI titers. P-values were calculated using the Mann Whitney rank test in GraphPad Prism 6. For correlation analyses the data for age, time of sample collection, HI titers and fold change were rank ordered and the Pearson product moment correlation coefficients were calculated in excel.

## Electronic supplementary material

Supplementary information.
