# Supplementary material for: Different Repeat Annual Influenza Vaccinations Improve the Antibody Response to Drifted Influenza Strains
Source: Sci Rep. 2017 Jul 12;7:5258. doi: 10.1038/s41598-017-05579-4 (PMC5507920; doi:10.1038/s41598-017-05579-4)

Supplementary Information

Different Repeat Annual Influenza Vaccinations Improve the Antibody Response to Drifted Influenza Strains

Ewan P Plant*, Lucy J Fredell, Blake A Hatcher, Xing Li, Meng-Jung Chiang, Martina Kosikova, Hang Xie, Olga Zoueva, Angelia A Cost, Zhiping Ye, and Michael J Cooper

* Corresponding author: [ewan.plant@fda.hhs.gov](mailto:ewan.plant@fda.hhs.gov), +1 240 402-7319

| **Antigen** | **V1** | **V2** | **Group** | **Correlation** | **Significance** |
| --- | --- | --- | --- | --- | --- |
| **H1N1 A/Beijing/262/1995** | + | + | Drift Identical | -0.140 | 0.22 |
|  | + | - | Drift Differing | -0.112 | 0.32 |
| **H1N1 A/New Caledonia/20/1999** | - | - | Drift Identical | -0.049 | 0.67 |
|  | - | + | Drift Differing | -0.052 | 0.65 |
|  | + | + | B Identical | -0.127 | 0.26 |
|  | + | + | B Differing | 0.000 | 0.99 |
| **H1N1 A/Brisbane/59/2007** | - | - | Shift Identical | 0.135 | 0.23 |
|  | + | - | Shift Differing | -0.129 | 0.26 |
|  | - | - | Drift Identical | -0.091 | 0.42 |
|  | - | - | Drift Differing | -0.026 | 0.82 |
| **H1N1 A/California/07/2009** | + | + | Shift Identical | -0.097 | 0.39 |
|  | - | + | Shift Differing | -0.049 | 0.66 |
|  | - | - | Drift Identical | 0.012 | 0.92 |
|  | - | - | Drift Differing | 0.151 | 0.18 |
| **H3N2 A/Sydney/5/1997** | + | + | Drift Identical | -0.105 | 0.35 |
|  | + | - | Drift Differing | -0.130 | 0.25 |
| **H3N2 A/Moscow/10/1999** | - | - | Drift Identical | -0.089 | 0.43 |
|  | - | + | Drift Differing | -0.056 | 0.62 |
|  | + | + | B Identical | -0.090 | 0.43 |
|  | + | + | B Differing | 0.108 | 0.34 |
| **H3N2 A/Wyoming/03/2003** | - | - | Drift Identical | 0.000 | 0.99 |
|  | - | - | Drift Differing | -0.087 | 0.44 |
| **H3N2 A/Perth/16/2009** | + | + | Shift Identical | -0.110 | 0.33 |
|  | - | + | Shift Differing | 0.056 | 0.62 |
|  | - | - | Drift Identical | 0.065 | 0.57 |
|  | - | - | Drift Differing | -0.002 | 0.99 |
| **B/Beijing/184/1993 (Victoria)** | + | + | Drift Identical | -0.047 | 0.68 |
|  | + | + | Drift Differing | -0.060 | 0.60 |
| **B/Sichuan/379/1999 (Yamagata)** | - | - | B Identical | -0.099 | 0.38 |
|  | + | - | B Differing | 0.228 | 0.042 |
| **B/Hong Kong/330/2001 (Victoria)** | + | + | B Identical | -0.126 | 0.27 |
|  | - | + | B Differing | -0.162 | 0.15 |
| **B/Brisbane/60/2008 (Victoria)** | + | + | Shift Identical | -0.280 | 0.011 |
|  | + | + | Shift Differing | -0.065 | 0.57 |
|  | - | - | Drift Identical | -0.179 | 0.11 |
|  | - | - | Drift Differing | -0.187 | 0.097 |
|  | - | - | B Identical | -0.116 | 0.30 |
|  | - | - | B Differing | -0.144 | 0.20 |
| **B/Massachusetts/02/2012 (Yamagata)** | - | - | B Identical | -0.206 | 0.067 |
|  | - | - | B Differing | 0.190 | 0.092 |

Supplementary Table 1. Correlation between Post-Vaccination GMT and Timing of Blood Draw. The Pearson product moment correlation coefficients for the timing of the post-vaccination blood draw and GMT values and associated p-values are shown.

| **Antigen** | **Group** | **Age**  **Correlation** | **Significance** | **Fold-Change**  **Correlation** | **Significance** |
| --- | --- | --- | --- | --- | --- |
| **H1N1 A/Beijing/262/1995** | Drift Identical | -0.155 | 0.17 | -0.055 | 0.63 |
|  | Drift Differing | -0.137 | 0.23 | -0.238 | 0.033 |
| **H1N1 A/New Caledonia/20/1999** | Drift Identical | -0.241 | 0.031 | -0.202 | 0.072 |
|  | Drift Differing | -0.227 | 0.043 | -0.222 | 0.048 |
|  | B Identical | -0.317 | 0.004 | -0.124 | 0.27 |
|  | B Differing | -0.519 | 8E-7 | -0.266 | 0.017 |
| **H1N1 A/Brisbane/59/2007** | Shift Identical | 0.079 | 0.95 | 0.130 | 0.25 |
|  | Shift Differing | -0.097 | 0.39 | -0.290 | 0.009 |
|  | Drift Identical | -0.031 | 0.79 | -0.058 | 0.61 |
|  | Drift Differing | -0.054 | 0.64 | -0.023 | 0.84 |
| **H1N1 A/California/07/2009** | Shift Identical | -0.249 | 0.026 | -0.106 | 0.39 |
|  | Shift Differing | -0.282 | 0.011 | -0.253 | 0.024 |
|  | Drift Identical | -0.092 | 0.42 | -0.019 | 0.87 |
|  | Drift Differing | -0.128 | 0.26 | -0.153 | 0.17 |
| **H3N2 A/Sydney/5/1997** | Drift Identical | -0.380 | 0.0005 | 0.003 | 0.98 |
|  | Drift Differing | -0.215 | 0.056 | -0.089 | 0.43 |
| **H3N2 A/Moscow/10/1999** | Drift Identical | -0.339 | 0.002 | -0.155 | 0.17 |
|  | Drift Differing | -0.173 | 0.13 | 0.017 | 0.88 |
|  | B Identical | -0.117 | 0.30 | -0.188 | 0.095 |
|  | B Differing | -0.310 | 0.005 | -0.151 | 0.18 |
| **H3N2 A/Wyoming/03/2003** | Drift Identical | -0.306 | 0.006 | -0.181 | 0.11 |
|  | Drift Differing | -0.120 | 0.29 | -0.005 | 0.97 |
| **H3N2 A/Perth/16/2009** | Shift Identical | -0.150 | 0.18 | -0.007 | 0.95 |
|  | Shift Differing | -0.067 | 0.56 | -0.064 | 0.57 |
|  | Drift Identical | -0.060 | 0.60 | -0.020 | 0.86 |
|  | Drift Differing | 0.097 | 0.39 | 0.101 | 0.37 |
| **B/Beijing/184/1993 (Victoria)** | Drift Identical | -0.277 | 0.014 | -0.070 | 0.54 |
|  | Drift Differing | -0.110 | 0.33 | -0.092 | 0.42 |
| **B/Sichuan/379/1999 (Yamagata)** | B Identical | -0.078 | 0.49 | 0.087 | 0.45 |
|  | B Differing | -0.286 | 0.01 | -0.019 | 0.87 |
| **B/Hong Kong/330/2001 (Victoria)** | B Identical | 0.058 | 0.61 | 0.117 | 0.30 |
|  | B Differing | -0.142 | 0.21 | -0.124 | 0.27 |
| **B/Brisbane/60/2008 (Victoria)** | Shift Identical | 0.335 | 0.002 | -0.032 | 0.78 |
|  | Shift Differing | 0.015 | 0.89 | -0.107 | 0.34 |
|  | Drift Identical | -0.112 | 0.32 | -0.016 | 0.89 |
|  | Drift Differing | 0.325 | 0.003 | 0.140 | 0.21 |
|  | B Identical | 0.193 | 0.086 | 0.112 | 0.32 |
|  | B Differing | -0.326 | 0.003 | -0.113 | 0.32 |
| **B/Massachusetts/02/2012 (Yamagata)** | B Identical | -0.142 | 0.21 | 0.120 | 0.29 |
|  | B Differing | -0.037 | 0.75 | -0.114 | 0.31 |

Supplementary Table 2. Correlations between Post-Vaccination GMT, Fold-Change and Age. The Pearson product moment correlation coefficients for the GMT values and fold-change with age and associated p-values are shown.

| **Antigen** | **V1** | **V2** | **Group** | **GMT Men** | **GMT Women** |
| --- | --- | --- | --- | --- | --- |
| **H1N1 A/Beijing/262/1995** | + | + | Drift Identical | 237 | **202** |
|  | + | - | Drift Differing | 859 | **710** |
| **H1N1 A/New Caledonia/20/1999** | - | - | Drift Identical | 67 | **36** |
|  | - | + | Drift Differing | 210 | **172** |
|  | + | + | B Identical | 177 | 199 |
|  | + | + | B Differing | 150 | ***71** |
| **H1N1 A/Brisbane/59/2007** | - | - | Shift Identical | 85 | 89 |
|  | + | - | Shift Differing | 164 | **136** |
|  | - | - | Drift Identical | 14 | 15 |
|  | - | - | Drift Differing | 59 | 75 |
| **H1N1 A/California/07/2009** | + | + | Shift Identical | 323 | 338 |
|  | - | + | Shift Differing | 191 | **87** |
|  | - | - | Drift Identical | 23 | **17** |
|  | - | - | Drift Differing | 49 | **37** |
| **H3N2 A/Sydney/5/1997** | + | + | Drift Identical | 237 | 254 |
|  | - | + | Drift Differing | 313 | 422 |
| **H3N2 A/Moscow/10/1999** | - | - | Drift Identical | 138 | 173 |
|  | - | + | Drift Differing | 215 | 243 |
|  | + | + | B Identical | 220 | **172** |
|  | + | + | B Differing | 200 | **160** |
| **H3N2 A/Wyoming/03/2003** | - | - | Drift Identical | 46 | **43** |
|  | - | - | Drift Differing | 70 | 102 |
| **H3N2 A/Perth/16/2009** | + | + | Shift Identical | 96 | **61** |
|  | - | + | Shift Differing | 50 | 56 |
|  | - | - | Drift Identical | 13 | **12** |
|  | - | - | Drift Differing | 12 | 13 |
| **B/Beijing/184/1993** | + | + | Drift Identical | 81 | **59** |
|  | + | + | Drift Differing | 102 | ***39** |
| **B/Sichuan/379/1999** | - | - | B Identical | 331 | **239** |
|  | + | - | B Differing | 327 | **198** |
| **B/Hong Kong/330/2001** | + | + | B Identical | 171 | **100** |
|  | - | + | B Differing | 164 | 202 |
| **B/Brisbane/60/2008** | + | + | Shift Identical | 301 | 320 |
|  | + | + | Shift Differing | 332 | 311 |
|  | - | - | Drift Identical | 57 | 57 |
|  | - | - | Drift Differing | 104 | ***39** |
|  | - | - | B Identical | 194 | **96** |
|  | - | - | B Differing | 259 | 296 |
| **B/Massachusetts/02/2012** | - | - | B Identical | 210 | **192** |
|  | - | - | B Differing | 289 | **244** |

Supplementary Table 3. GMT Titers Stratified by Sex. Significant differences in GMT between men and women are indicated by a single asterisk for p ≤ 0.05. The lower GMT values for women are in bold.

Supplementary Figure 1.

Reverse Cumulative Distribution Curves. The results for the pandemic shift cohorts are displayed. The number of samples is displayed on the y-axis and the titer is displayed on the x-axis. Titers for both pre- and post-vaccination samples for both the identical and differing groups are shown. Each antigen is displayed as a separate graph.


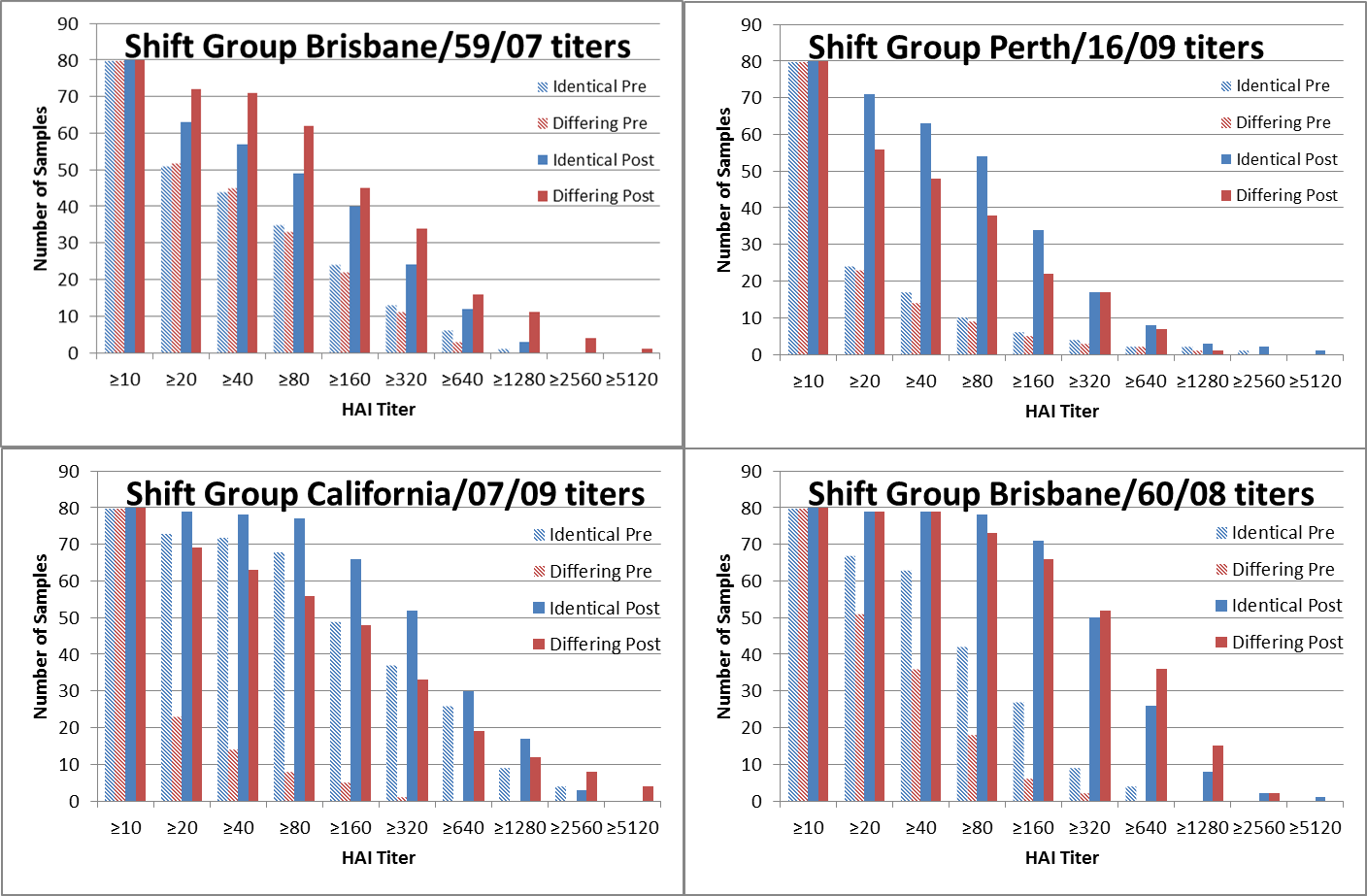


Supplementary Figure 2a.

The geometric mean titers for the influenza B viruses in the drift experimental group are shown with standard deviations. The significance of the difference between the identical and differing groups using the Mann Whitney test is shown. ** p ≤ 0.01.


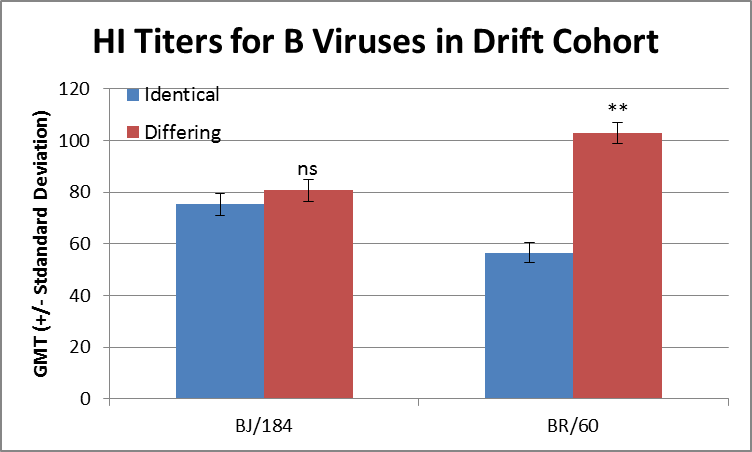


Supplementary Figure 2b.

Reverse Cumulative Distribution Curves. The results for the H1N1 viruses in the drift cohorts are displayed. The number of samples is displayed on the y-axis and the titer is displayed on the x-axis. Titers for both pre- and post-vaccination samples for both the identical and differing groups are shown. The antigens included in the vaccines are shown in the graphs on the left and the antigens that emerged in later years are shown on the right.


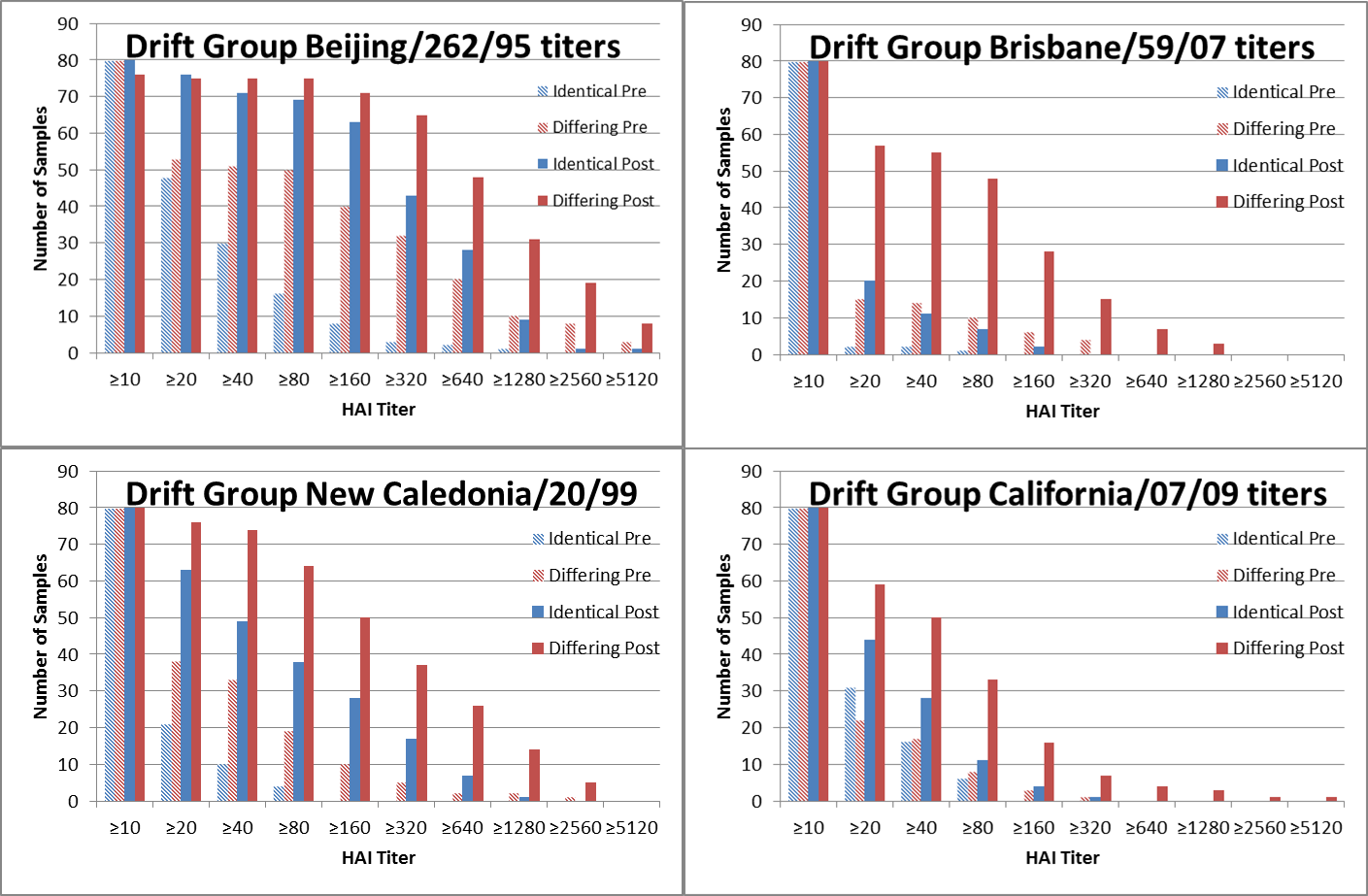


Supplementary Figure 2c.

Reverse Cumulative Distribution Curves. The results for the H3N2 viruses in the drift cohorts are displayed. The number of samples is displayed on the y-axis and the titer is displayed on the x-axis. Titers for both pre- and post-vaccination samples for both the identical and differing groups are shown. The antigens included in the vaccines are shown in the graphs on the left and the antigens that emerged in later years are shown on the right.


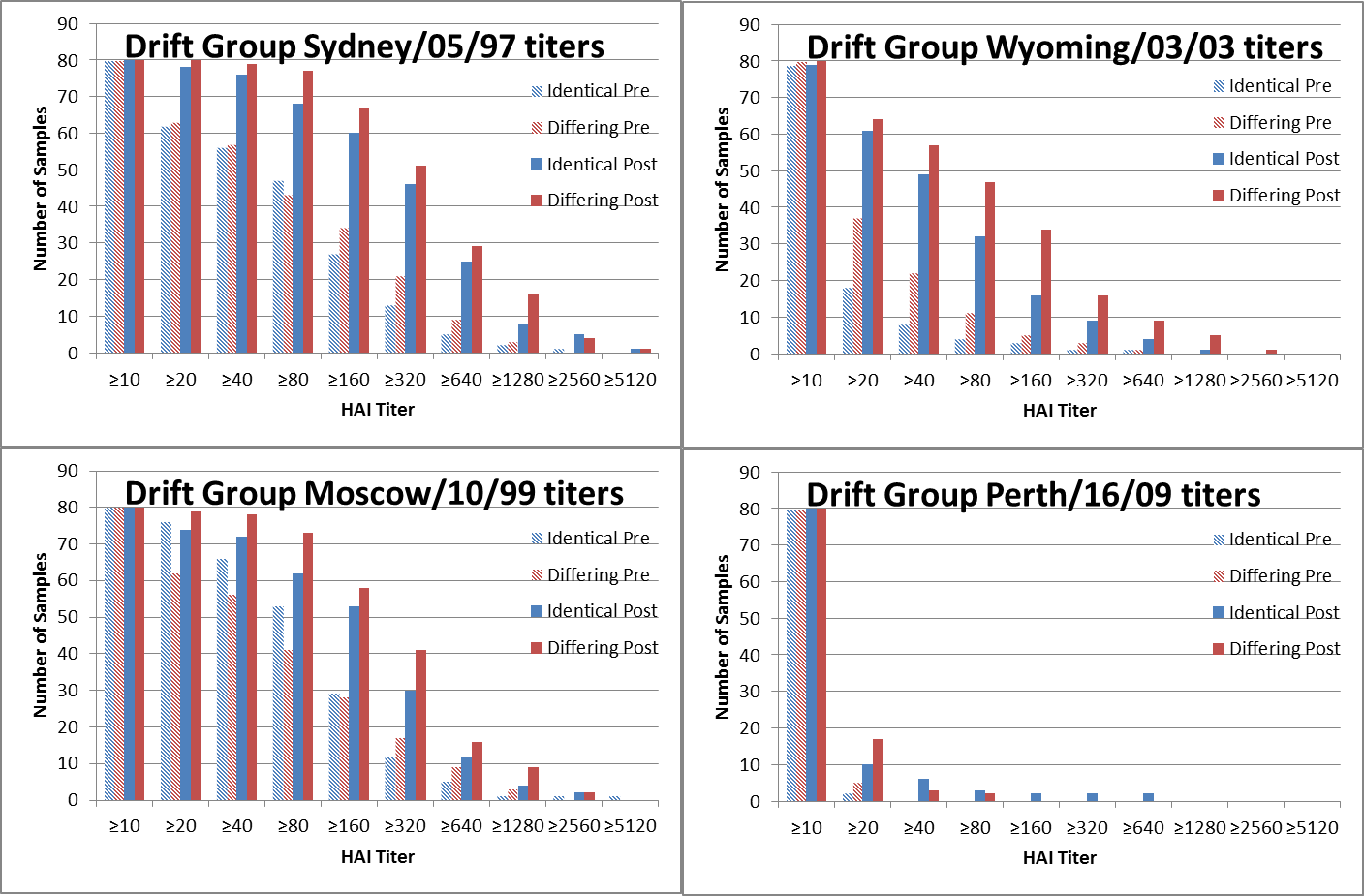


Supplementary Figure 2d.

Reverse Cumulative Distribution Curves. The results for the B viruses in the drift cohorts are displayed. The number of samples is displayed on the y-axis and the titer is displayed on the x-axis. Titers for both pre- and post-vaccination samples for both the identical and differing groups are shown. Each antigen is displayed as a separate graph.


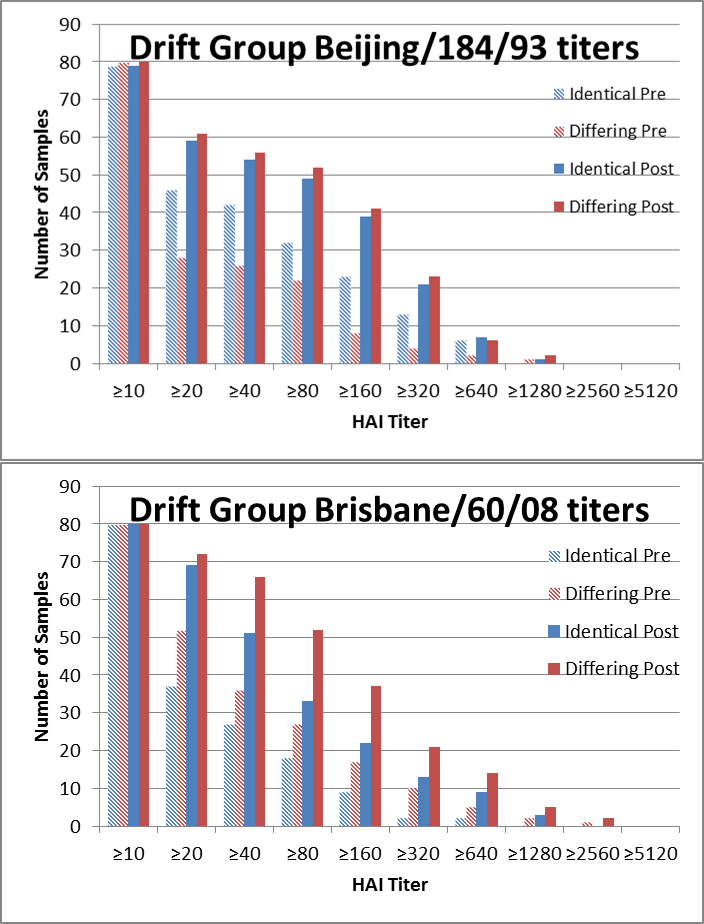


Supplementary Figure 3a.

The geometric mean titers for the B virus experimental group are shown with standard deviations. The significance of the difference between the identical and differing groups using the Mann Whitney test is shown. * p ≤ 0.05, *** p ≤ 0.001.


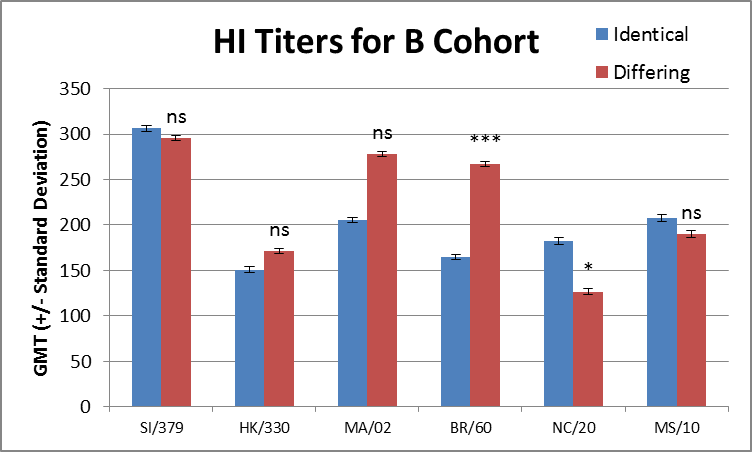


Supplementary Figure 3b.

Reverse Cumulative Distribution Curves. The results for the B viruses in the B virus cohorts are displayed. The number of samples is displayed on the y-axis and the titer is displayed on the x-axis. Titers for both pre- and post-vaccination samples for both the identical and differing groups are shown. The antigens included in the vaccines are shown in the graphs on the left and the antigens that emerged in later years are shown on the right.


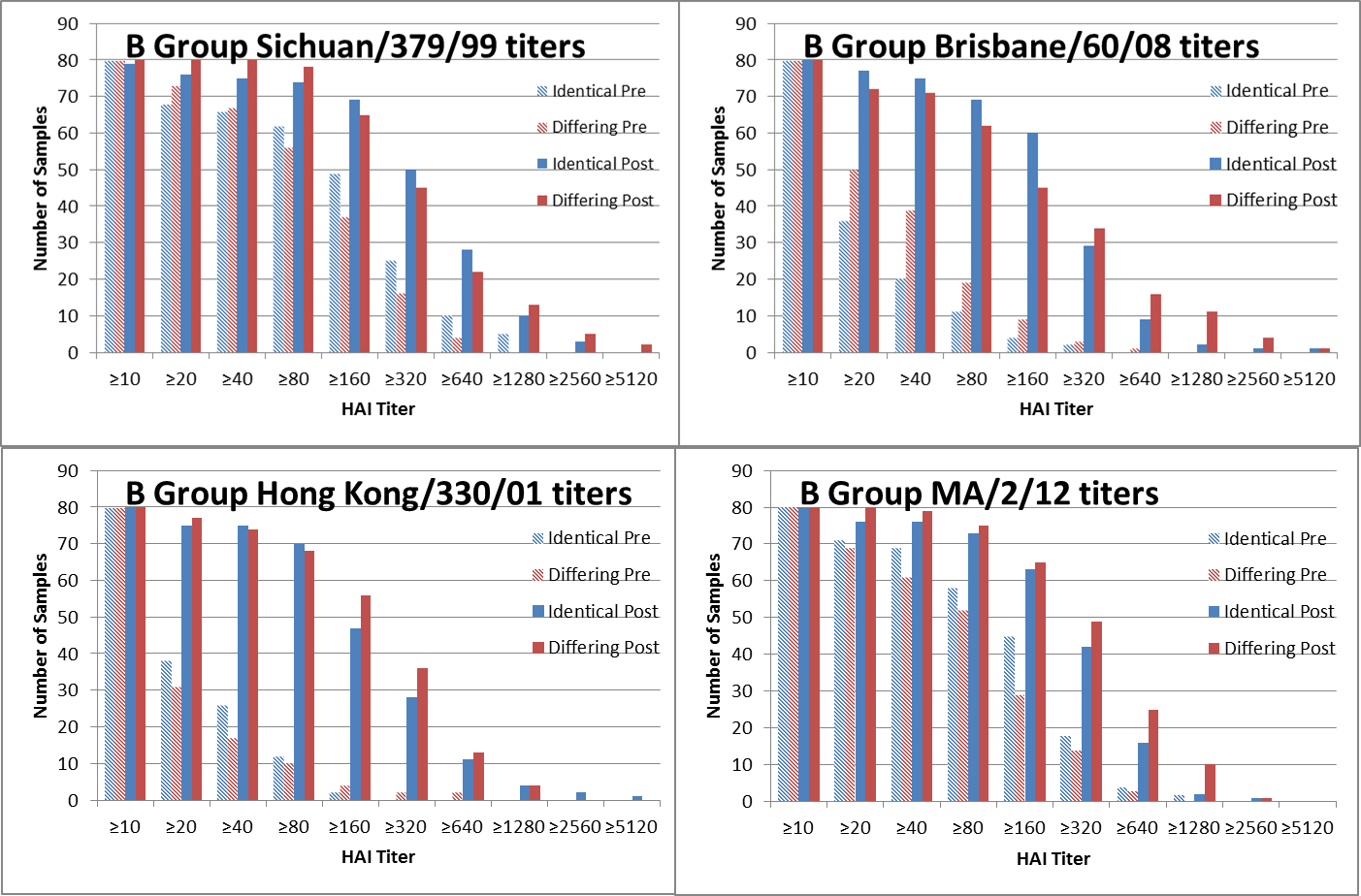


Supplementary Figure 3c.

Reverse Cumulative Distribution Curves. The results for the influenza A viruses in the B virus cohorts are displayed. The number of samples is displayed on the y-axis and the titer is displayed on the x-axis. Titers for both pre- and post-vaccination samples for both the identical and differing groups are shown. Each antigen is displayed as a separate graph.


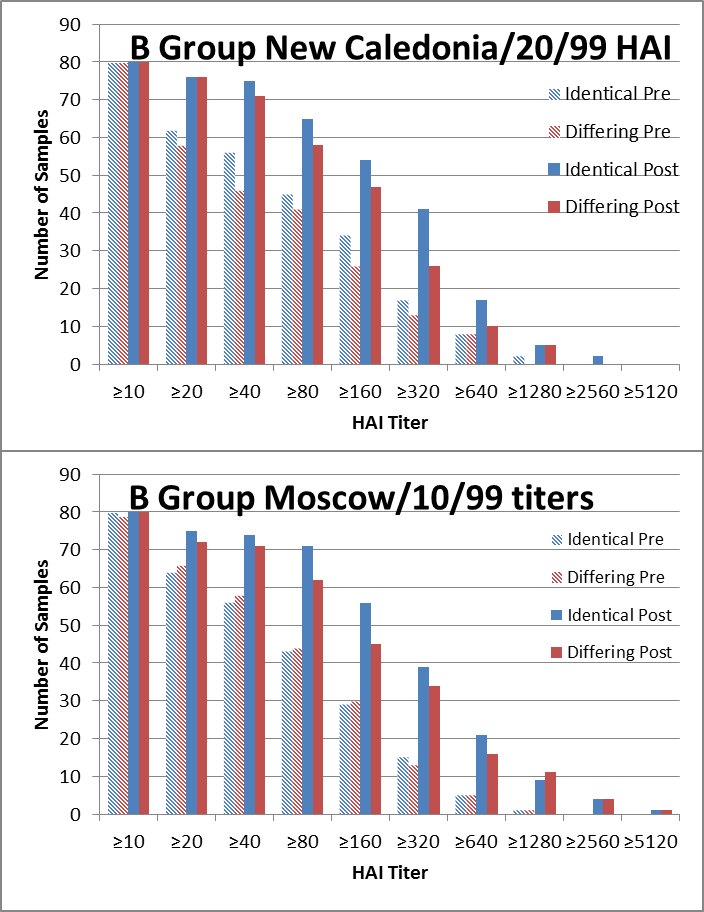


Supplementary Figure 4.

The geometric mean titers for all experimental groups with data from subjects with medically attended ILI or PI removed are graphed. The titer is displayed on the y-axis and the antigen on the x-axis. Graphs for the pandemic shift and influenza B virus cohorts are shown on the left and graphs for the drift cohorts are shown on the right. The significance of the difference between the identical and differing groups using the Mann Whitney test is shown. * p ≤ 0.05, ** p ≤ 0.01 and *** p ≤ 0.001.


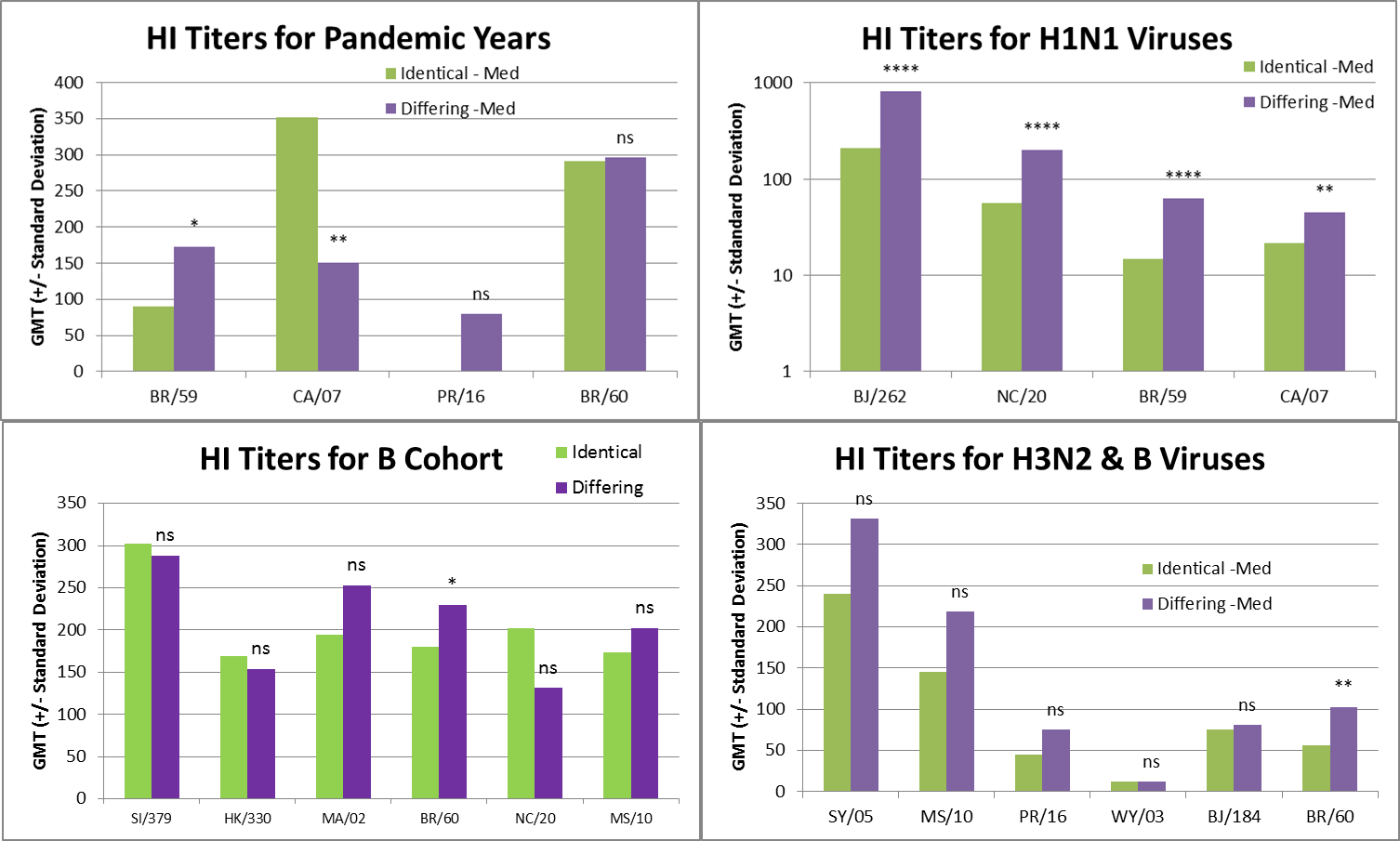

Supplement: Supplementary file 1 — Supplementary information. [file 41598_2017_5579_MOESM1_ESM.doc]
